# Radiogenomics correlation between MR imaging features and mRNA-based subtypes in lower-grade glioma

**DOI:** 10.1186/s12883-020-01838-6

**Published:** 2020-06-29

**Authors:** Zhenyin Liu, Jing Zhang

**Affiliations:** Department of Medical Imaging, Guangzhou Women and Children’s Medical Center, Guangzhou Medical University, 9 Jinsui Road, Guangzhou City, 510623 PR China

**Keywords:** Logistic regression, Lower-grade gliomas, mRNA-based subtypes, MR biomarker, Time-dependent ROC

## Abstract

**Background:**

To investigate associations between lower-grade glioma (LGG) mRNA-based subtypes (R1-R4) and MR features.

**Methods:**

mRNA-based subtyping was obtained from the LGG dataset in The Cancer Genome Atlas (TCGA). We identified matching patients (*n* = 145) in The Cancer Imaging Archive (TCIA) who underwent MR imaging. The associations between mRNA-based subtypes and MR features were assessed.

**Results:**

In the TCGA-LGG dataset, patients with the R2 subtype had the shortest median OS months (*P* < 0.05). The time-dependent ROC for the R2 subtype was 0.78 for survival at 12 months, 0.76 for survival at 24 months, and 0.76 for survival at 36 months. In the TCIA-LGG dataset, 41 (23.7%) R1 subtype, 40 (23.1%) R2 subtype, 19 (11.0%) R3 subtype and 45 (26.0%) R4 subtype cases were identified. Multivariate analysis revealed that enhancing margin (ill-defined, OR: 9.985; *P* = 0.003) and T1 + C/T2 mismatch (yes, OR: 0.091; *P* = 0.023) were associated with the R1 subtype (AUC: 0.708). The average accuracy of the ten-fold cross validation was 71%. Proportion of contrast-enhanced (CE) tumour (> 5%, OR: 14.733; *P* < 0.001) and necrosis/cystic changes (yes, OR: 0.252; *P* = 0.009) were associated with the R2 subtype (AUC: 0.832). The average accuracy of the ten-fold cross validation was 82%. Haemorrhage (yes, OR: 8.55; *P* < 0.001) was positively associated with the R3 subtype (AUC: 0.689). The average accuracy of the ten-fold cross validation was 87%. Proportion of CE tumour (> 5%, OR: 0.14; *P* < 0.001) was negatively associated with the R4 subtype (AUC: 0.672). The average accuracy of the ten-fold cross validation was 71%. For the prediction of the R2 subtype, the nomogram showed good discrimination and calibration. Decision curve analysis demonstrated that prediction with the R2 model was clinically useful.

**Conclusions:**

Patients with the R2 subtype had the worst prognosis. We demonstrated that MRI features can identify distinct LGG mRNA-based molecular subtypes.

## Background

Primary brain tumours are one of the top ten causes of cancer-related deaths in the United States [1]. They are characterized by biological heterogeneity and can be classified into a variety of histological subtypes [2–4]. LGGs are currently classified by morphological criteria. However, this classification suffers from high interobserver and intraobserver variability [5, 6]. Therefore, clinicians increasingly rely on genetic classification to guide clinical decision making. The treatment of LGG could benefit from the incorporation of precision medicine. The majority of patients with high-risk LGG are treated with single-agent temozolomide (TMZ) and radiotherapy. Next-generation sequencing has definitively revealed that different LGG mRNA-based subtypes fundamentally differ in their underlying molecular pathways, despite being histologically similar [7–9].

A new direction in cancer research has emerged that focuses on the relationship between genomic data and imaging features [10–12]. Radiogenomic studies have indicated key imaging differences between certain LGG genetic groups and may aid in the diagnosis of LGG as well as the longitudinal assessment of treatment response and evaluation of tumour recurrence in patients with LGG [13–16]. The TCGA Research Network identifies four mRNA-based (R1–R4) subtypes [17] (N Engl J Med 2015). Core members in the four well-defined subtypes were identified and found to be distinctly enriched for the previously defined astrocytoma subtype and neural ontology signatures and correlated with specific genomic events.

Survival analysis revealed that the R2 subtype was significantly correlated with shorter overall survival. Both the R1 subtype and R3 subtype highly expressed an early progenitor-like astrocytoma gene signature. The R4 subtype highly expressed a neuroblastic astrocytoma signature and a neuron-specific signature [17]. This study aims to explore associations between LGG mRNA-based subtypes (R1-R4) and MR features. Our preliminary radiogenomics analysis may serve as a reference in the development of precision medicine for LGG patients.

## Methods

### Patient population

The clinical files of LGG patients were obtained from TCGA. MR data were provided by TCIA [18–20]. TCGA and TCIA are publicly available databases. The TCGA Research Network [17] classifies LGG into four categories (R1, R2, R3 and R4) according to mRNA expression patterns. The inclusion criteria of the study were as follows: (I) mRNA-based subtyping (R1-R4) was obtained from the LGG dataset in TCGA; and (II) MR data were available from TCIA (T1WI, T2WI, contrast enhancement). Unevaluable examinations and postsurgical patients were excluded. Finally, 145 patients met the inclusion criteria.

### Assessment of MR features

MR image analysis was performed as previously published by recent studies and our group [21–23]. The following lesion features were evaluated [21–23]: (A) volume (<median or > =median); (B) width (<median or > =median); (C) length (<median or > =median); (D) depth (<median or > =median); (E) proportion of CE tumour (no [<=5%] or yes [> 5%]); (F) enhancing margin (well-defined or poorly defined); (G) T1 + C/T2 mismatch (negative or positive); (H) extranodular growth (negative or positive); (I) shortest distance between the tumour centroid and the lateral edge [CS](<=30 mm or > 30 mm); (J) subventricular zone [SVZ] involvement (negative or positive); (K) location (frontal lobe or other); (XII) volume (> = 60 cm^3^ or < 60 cm^3^); (L) haemorrhage (negative or positive); (M) multifocal (negative or positive); (N) degree of enhancement (slight or obvious); and (O) necrosis/cystic change (negative or positive). Both neuroradiologists were blinded to LGG mRNA-based subtypes (R1-R4) as well as the clinical data.

### Statistical analysis

We focused on the association of mRNA-based subtypes (R1-R4) and MR features. A colour heat map was drawn to show the correlation patterns between MR features and mRNA expression (R1-R4). Fisher’s exact test, the chi-square test and binary logistic regression analysis were used (version 23.0; SPSS Company) for each mRNA-based subtype. We use tenfold cross-validation test. Odds ratios (ORs) as well as their corresponding 95% confidence intervals (CIs) are reported. In the present study, binary logistic regression was repeated for the four LGG mRNA-based subtypes: R1, R2, R3 and R4. The area under the receiver operating characteristic curve (AUC) of each mRNA-based subtype (R1-R4) is reported. Survival analysis was conducted by using Kaplan-Meier analysis and the time-dependent ROC method (the worst prognostic subgroup; R package). A *P*-value of less than 0.05 (two-sided) was considered to indicate statistical significance.

## Results

**Fig. 1:**
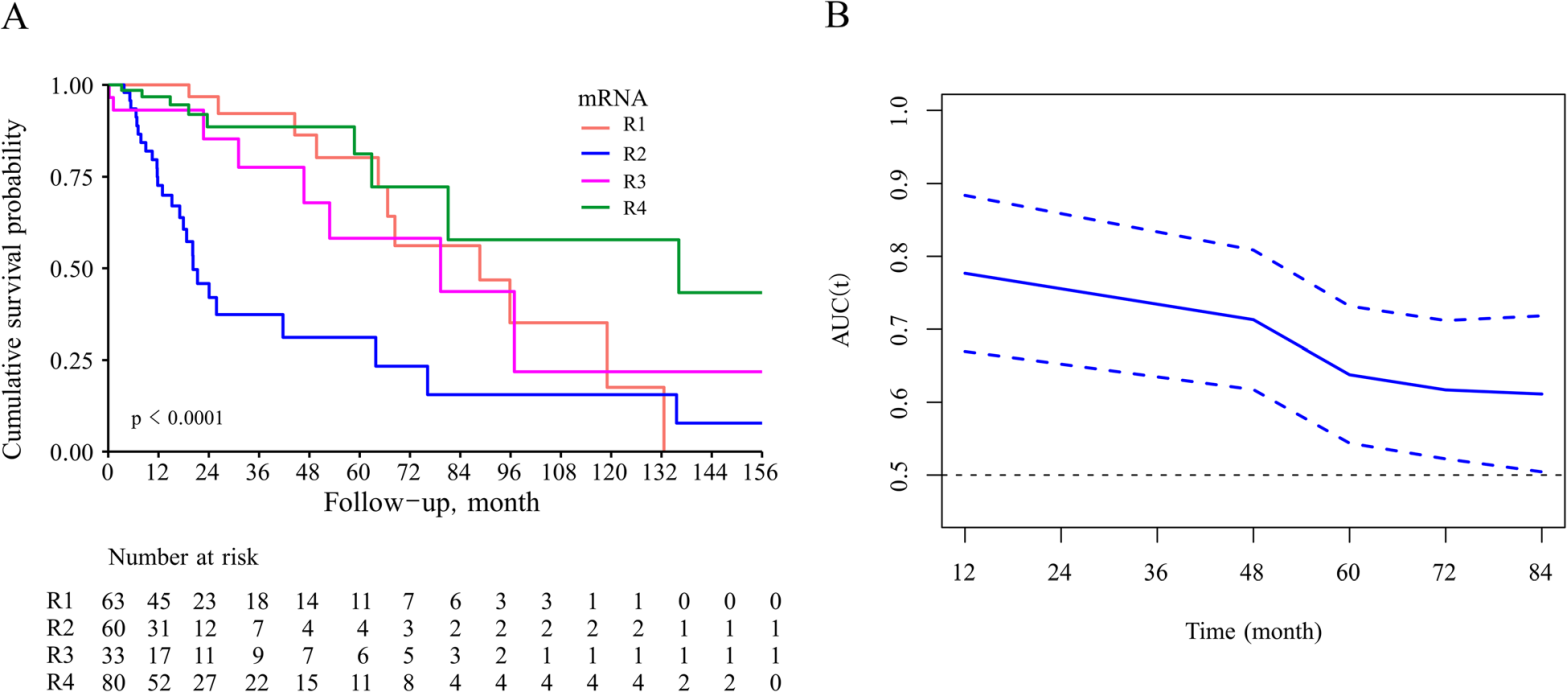
Kaplan-Meier curve (**a**) and time-dependent ROC curve (**b**) for OS between the R2-subtype and non-R2-subtype groups

In the TCGA-LGG dataset, patients with the R2 subtype had the shortest median OS months (*P* < 0.05 Fig. 1a). The time-dependent ROC for the R2 subtype was 0.78 for survival at 12 months, 0.76 for survival at 24 months, and 0.76 for survival at 36 months (Fig. 1b).

**Fig. 2:**
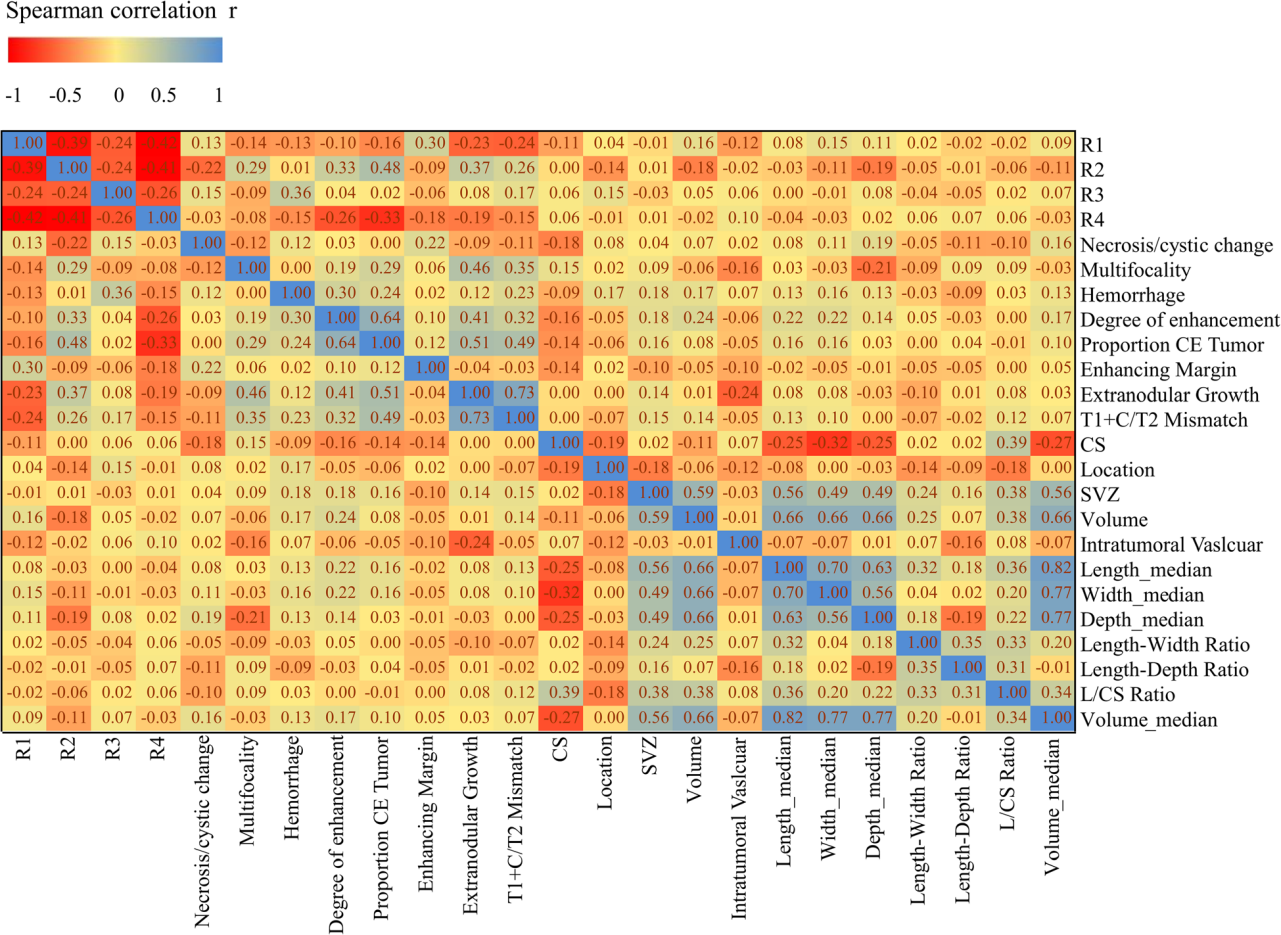
The pairwise Spearman’s rank correlation coefficients of the qualitative MR features and mRNA-based subtypes (R1-R4)

In the TCIA-LGG dataset, a total of 145 LGG (grade II: 61 cases, grade III 68 cases) patients (IDHmut-non-codeletion: 70 cases; IDHmut-codeletion: 35 cases; IDHwt: 38 cases) were screened (female: 70 patients; male: 75 patients). The demographic and tumour characteristics of all 145 patients (astrocytoma: 49 cases; oligoastrocytoma: 31 cases; oligodendroglioma: 65 cases) are summarized in Table 1. The identified subtypes were as follows: 41 (23.7%) R1 subtype, 40 (23.1%) R2 subtype, 19 (11.0%) R3 subtype and 45 (26.0%) R4 subtype. The frequencies (heat map) of lesion features per mRNA-based subtype (R1-R4) are pictured in Fig. 2.

**Table 1:** Clinical Characteristics of the LGG Sample Set According to RNA subtypes

	Total (*N* = 145)	RNAR1		RNAR2		RNAR3		RNAR4	
		(−)	(+)	(−)	(+)	(−)	(+)	(−)	(+)
Histological type — no. (%)									
Oligodendroglioma	65 (44.83)	51 (49.04)	14 (34.15)	58 (55.24)	7 (17.5)	49 (38.89)	16 (84.21)	37 (37)	28 (62.22)
Astrocytoma	49 (33.79)	36 (34.62)	13 (31.71)	21 (20)	28 (70)	48 (38.1)	1 (5.26)	42 (42)	7 (15.56)
Oligoastrocytoma	31 (21.38)	17 (16.35)	14 (34.15)	26 (24.76)	5 (12.5)	29 (23.02)	2 (10.53)	21 (21)	10 (22.22)
Neoplasm histologic grade — no. (%)									
G2	71 (48.97)	42 (40.38)	29 (70.73)	64 (60.95)	7 (17.5)	64 (50.79)	7 (36.84)	43 (43)	28 (62.22)
G3	74 (51.03)	62 (59.62)	12 (29.27)	41 (39.05)	33 (82.5)	62 (49.21)	12 (63.16)	57 (57)	17 (37.78)
Age at diagnosis— yr									
Mean	44.06 ± 13.78	46.46 ± 13.69	37.98 ± 12.18	41.83 ± 13.09	49.93 ± 13.98	42.72 ± 13.63	52.95 ± 11.53	45.6 ± 14.25	40.64 ± 12.15
Range	18–75	20–75	18–70	18–75	23–70	18–70	30–75	18–75	20–67
Gender — no. (%)									
Female	70 (48.28)	52 (50)	18 (43.9)	49 (46.67)	21 (52.5)	61 (48.41)	9 (47.37)	48 (48)	22 (48.89)
Male	75 (51.72)	52 (50)	23 (56.1)	56 (53.33)	19 (47.5)	65 (51.59)	10 (52.63)	52 (52)	23 (51.11)
Race — no. (%)									
American Indian or Alaska Native	0 (0)	0 (0)	0 (0)	0 (0)	0 (0)	0 (0)	0 (0)	0 (0)	0 (0)
Black or African American	8 (5.56)	5 (4.81)	3 (7.5)	6 (5.77)	2 (5)	8 (6.4)	0 (0)	5 (5.05)	3 (6.67)
White	136 (94.44)	99 (95.19)	37 (92.5)	98 (94.23)	38 (95)	117 (93.6)	19 (100)	94 (94.95)	42 (93.33)
Family history of cancer — no. (%)									
No	60 (55.05)	37 (48.05)	23 (71.88)	47 (58.02)	13 (46.43)	55 (56.7)	5 (41.67)	41 (56.94)	19 (51.35)
Yes	49 (44.95)	40 (51.95)	9 (28.13)	34 (41.98)	15 (53.57)	42 (43.3)	7 (58.33)	31 (43.06)	18 (48.65)
Method of sample procuremen — no. (%)									
Open biopsy	5 (3.45)	4 (3.85)	1 (2.44)	3 (2.86)	2 (5)	5 (3.97)	0 (0)	3 (3)	2 (4.44)
Subtotal resection	45 (31.03)	33 (31.73)	12 (29.27)	30 (28.57)	15 (37.5)	38 (30.16)	7 (36.84)	34 (34)	11 (24.44)
Gross total resection	95 (65.52)	67 (64.42)	28 (68.29)	72 (68.57)	23 (57.5)	83 (65.87)	12 (63.16)	63 (63)	32 (71.11)
First presenting symptom — no. (%)									
Headaches	35 (25)	20 (19.8)	15 (38.46)	28 (27.72)	7 (17.95)	32 (26.23)	3 (16.67)	25 (26.04)	10 (22.73)
Mental status change	11 (7.86)	9 (8.91)	2 (5.13)	8 (7.92)	3 (7.69)	8 (6.56)	3 (16.67)	8 (8.33)	3 (6.82)
Motor or movement change	9 (6.43)	8 (7.92)	1 (2.56)	6 (5.94)	3 (7.69)	7 (5.74)	2 (11.11)	6 (6.25)	3 (6.82)
Seizure	79 (56.43)	58 (57.43)	21 (53.85)	55 (54.46)	24 (61.54)	71 (58.2)	8 (44.44)	53 (55.21)	26 (59.09)
Sensory or visual change	6 (4.29)	6 (5.94)	0 (0)	4 (3.96)	2 (5.13)	4 (3.28)	2 (11.11)	4 (4.17)	2 (4.55)
Laterality — no. (%)									
Left	70 (48.61)	49 (47.57)	21 (51.22)	51 (49.04)	19 (47.5)	63 (50.4)	7 (36.84)	47 (47)	23 (52.27)
Midline	3 (2.08)	3 (2.91)	0 (0)	1 (0.96)	2 (5)	2 (1.6)	1 (5.26)	3 (3)	0 (0)
Right	71 (49.31)	51 (49.51)	20 (48.78)	52 (50)	19 (47.5)	60 (48)	11 (57.89)	50 (50)	21 (47.73)
IDH/1p19q Subtype									
IDHmut-non-codel	70 (48.95)	31 (30.1)	39 (97.5)	59 (57.28)	11 (27.5)	70 (56)	0 (0)	50 (51.02)	20 (44.44)
IDHmut-codel	35 (24.48)	35 (33.98)	0 (0)	35 (33.98)	0 (0)	17 (13.6)	18 (100)	18 (18.37)	17 (37.78)
IDHwt	38 (26.57)	37 (35.92)	1 (2.5)	9 (8.74)	29 (72.5)	38 (30.4)	0 (0)	30 (30.61)	8 (17.78)

In univariate analysis (Tables 2 and 3), enhancing margin, extranodular growth and T1 + C/T2 mismatch were associated with the R1 subtype (all *P* < 0.05). Necrosis/cystic change, multifocality, degree of enhancement, proportion of CE tumour, volume, depth, extranodular growth and T1 + C/T2 mismatch were associated with the R2 subtype. Haemorrhage was associated with the R3 subtype (*P* < 0.05). The degree of enhancement, enhancing margin, proportion of CE tumour and extranodular growth were associated with the R4 subtype (all *P* < 0.05).

**Table 2:** Summary of MR features per mRNA-based subgroup

		R1		R2		R3		R4	
		Negative	Positive	Negative	Positive	Negative	Positive	Negative	Positive
Necrosis/cystic	Negative	25 (24.04)	5 (12.2)	16 (15.24)	14 (35)	29 (23.02)	1 (5.26)	20 (20)	10 (22.22)
	Positive	79 (75.96)	36 (87.8)	89 (84.76)	26 (65)	97 (76.98)	18 (94.74)	80 (80)	35 (77.78)
Multifocality	Negative	97 (93.27)	41 (100)	104 (99.05)	34 (85)	119 (94.44)	19 (100)	94 (94)	44 (97.78)
	Positive	7 (6.73)	0 (0)	1 (0.95)	6 (15)	7 (5.56)	0 (0)	6 (6)	1 (2.22)
Hemorrhage	Negative	86 (82.69)	38 (92.68)	90 (85.71)	34 (85)	114 (90.48)	10 (52.63)	82 (82)	42 (93.33)
	Positive	18 (17.31)	3 (7.32)	15 (14.29)	6 (15)	12 (9.52)	9 (47.37)	18 (18)	3 (6.67)
Degree of enhancement	Negative	38 (39.18)	20 (50)	52 (52.53)	6 (15.79)	51 (43.22)	7 (36.84)	33 (34.02)	25 (62.5)
	Positive	59 (60.82)	20 (50)	47 (47.47)	32 (84.21)	67 (56.78)	12 (63.16)	64 (65.98)	15 (37.5)
Proportion CE Tumor	Negative	59 (60.82)	31 (77.5)	79 (79.8)	11 (28.95)	78 (66.1)	12 (63.16)	54 (55.67)	36 (90)
	Positive	38 (39.18)	9 (22.5)	20 (20.2)	27 (71.05)	40 (33.9)	7 (36.84)	43 (44.33)	4 (10)
Enhancing Margin	Well-defined	33 (34.02)	2 (5)	23 (23.23)	12 (31.58)	29 (24.58)	6 (31.58)	20 (20.62)	15 (37.5)
	Poorly-defined	64 (65.98)	38 (95)	76 (76.77)	26 (68.42)	89 (75.42)	13 (68.42)	77 (79.38)	25 (62.5)
Extranodular Growth	Negative	73 (75.26)	38 (95)	89 (89.9)	22 (57.89)	97 (82.2)	14 (73.68)	74 (76.29)	37 (92.5)
	Positive	24 (24.74)	2 (5)	10 (10.1)	16 (42.11)	21 (17.8)	5 (26.32)	23 (23.71)	3 (7.5)
T1 + C/T2 Mismatch	Negative	76 (78.35)	39 (97.5)	89 (89.9)	26 (68.42)	102 (86.44)	13 (68.42)	78 (80.41)	37 (92.5)
	Positive	21 (21.65)	1 (2.5)	10 (10.1)	12 (31.58)	16 (13.56)	6 (31.58)	19 (19.59)	3 (7.5)
CS	> 30 mm	47 (46.53)	24 (58.54)	52 (50)	19 (50)	63 (51.22)	8 (42.11)	51 (52.04)	20 (45.45)
	<=30 mm	54 (53.47)	17 (41.46)	52 (50)	19 (50)	60 (48.78)	11 (57.89)	47 (47.96)	24 (54.55)
Location	Other	65 (62.5)	24 (58.54)	60 (57.14)	29 (72.5)	81 (64.29)	8 (42.11)	61 (61)	28 (62.22)
	Frontal lobe	39 (37.5)	17 (41.46)	45 (42.86)	11 (27.5)	45 (35.71)	11 (57.89)	39 (39)	17 (37.78)
SVZ	Negative	40 (38.46)	16 (39.02)	41 (39.05)	15 (37.5)	48 (38.1)	8 (42.11)	39 (39)	17 (37.78)
	Positive	64 (61.54)	25 (60.98)	64 (60.95)	25 (62.5)	78 (61.9)	11 (57.89)	61 (61)	28 (62.22)
Volume	<60cm^3^	38 (36.54)	8 (19.51)	28 (26.67)	18 (45)	41 (32.54)	5 (26.32)	31 (31)	15 (33.33)
	> = 60 cm^3^	66 (63.46)	33 (80.49)	77 (73.33)	22 (55)	85 (67.46)	14 (73.68)	69 (69)	30 (66.67)
Intratumoral vascular	Negative	1 (1.03)	2 (5)	2 (2.02)	1 (2.63)	3 (2.54)	0 (0)	3 (3.09)	0 (0)
	Positive	96 (98.97)	38 (95)	97 (97.98)	37 (97.37)	115 (97.46)	19 (100)	94 (96.91)	40 (100)
Length	<median	57 (54.81)	19 (46.34)	54 (51.43)	22 (55)	66 (52.38)	10 (52.63)	51 (51)	25 (55.56)
	> = median	47 (45.19)	22 (53.66)	51 (48.57)	18 (45)	60 (47.62)	9 (47.37)	49 (49)	20 (44.44)
Width	<median	58 (55.77)	16 (39.02)	50 (47.62)	24 (60)	64 (50.79)	10 (52.63)	50 (50)	24 (53.33)
	> = median	46 (44.23)	25 (60.98)	55 (52.38)	16 (40)	62 (49.21)	9 (47.37)	50 (50)	21 (46.67)
Depth	<median	58 (55.77)	18 (43.9)	49 (46.67)	27 (67.5)	68 (53.97)	8 (42.11)	53 (53)	23 (51.11)
	> = median	46 (44.23)	23 (56.1)	56 (53.33)	13 (32.5)	58 (46.03)	11 (57.89)	47 (47)	22 (48.89)
Length-Width Ratio	<median	56 (53.85)	21 (51.22)	54 (51.43)	23 (57.5)	66 (52.38)	11 (57.89)	55 (55)	22 (48.89)
	> = median	48 (46.15)	20 (48.78)	51 (48.57)	17 (42.5)	60 (47.62)	8 (42.11)	45 (45)	23 (51.11)
Length-Depth Ratio	<median	53 (50.96)	22 (53.66)	54 (51.43)	21 (52.5)	64 (50.79)	11 (57.89)	54 (54)	21 (46.67)
	> = median	51 (49.04)	19 (46.34)	51 (48.57)	19 (47.5)	62 (49.21)	8 (42.11)	46 (46)	24 (53.33)
L/CS Ratio	<median	50 (49.5)	21 (51.22)	50 (48.08)	21 (55.26)	62 (50.41)	9 (47.37)	51 (52.04)	20 (45.45)
	> = median	51 (50.5)	20 (48.78)	54 (51.92)	17 (44.74)	61 (49.59)	10 (52.63)	47 (47.96)	24 (54.55)
Volume	<median	56 (53.85)	18 (43.9)	50 (47.62)	24 (60)	66 (52.38)	8 (42.11)	50 (50)	24 (53.33)
	> = median	48 (46.15)	23 (56.1)	55 (52.38)	16 (40)	60 (47.62)	11 (57.89)	50 (50)	21 (46.67)

**Table 3:** Results from risk analyses (univariate logistic regression, odds ratios, 95% confidence intervals in parentheses)

		R1	R2	R3	R4
Necrosis/cystic	Negative	Reference			
	Positive	2.278 (0.807–6.433)	0.334 (0.144–0.773)*	5.381 (0.689–42.052)	0.875 (0.371–2.061)
Multifocality	Negative	Reference			
	Positive	–	18.353 (2.133–157.892)**	–	0.356 (0.042–3.048)
Hemorrhage	Negative	Reference			
	Positive	0.377 (0.105–1.357)	1.059 (0.38–2.953)	8.55 (2.906–25.158)***	0.325 (0.091–1.168)
Degree of enhancement	Negative	Reference			
	Positive	0.644 (0.307–1.352)	5.901 (2.266–15.365)***	1.305 (0.48–3.55)	0.309 (0.144–0.665)**
Proportion CE Tumor	Negative	Reference			
	Positive	0.451 (0.193–1.051)	9.695 (4.12–22.813)***	1.137 (0.416–3.114)	0.14 (0.046–0.423)***
Enhancing Margin	Well-defined	Reference			
	Poorly-defined	9.797 (2.224–43.151)**	0.656 (0.287–1.501)	0.706 (0.246–2.026)	0.433 (0.193–0.97)*
Extranodular Growth	Negative	Reference			
	Positive	0.16 (0.036–0.714)*	6.473 (2.586–16.203)***	1.65 (0.536–5.08)	0.261 (0.074–0.925)*
T1 + C/T2 Mismatch	Negative	Reference			
	Positive	0.093 (0.012–0.716)*	4.108 (1.595–10.58)**	2.942 (0.978–8.853)	0.333 (0.093–1.196)
CS	> 30 mm	Reference			
	<=30 mm	0.617 (0.296–1.285)	1 (0.476–2.102)	1.444 (0.544–3.835)	1.302 (0.638–2.658)
Location	Other	Reference			
	Frontal lobe	1.181 (0.565–2.468)	0.506 (0.229–1.119)	2.475 (0.928–6.601)	0.95 (0.46–1.959)
SVZ	Negative	Reference			
	Positive	0.977 (0.465–2.05)	1.068 (0.504–2.262)	0.846 (0.318–2.253)	1.053 (0.51–2.173)
Volume	<60cm^3^	Reference			
	> = 60 cm^3^	2.375 (0.996–5.666)	0.444 (0.208–0.949)*	1.351 (0.455–4.005)	0.899 (0.424–1.904)
Intratumoral vascular	Negative	Reference			
	Positive	0.198 (0.017–2.247)	0.763 (0.067–8.667)	266,904,432.61 (0–0)	687,436,275.335 (0–0)
Length	<median	Reference			
	> = median	1.404 (0.68–2.9)	0.866 (0.417–1.8)	0.99 (0.377–2.601)	0.833 (0.411–1.688)
Width	<median	Reference			
	> = median	1.97 (0.943–4.118)	0.606 (0.289–1.27)	0.929 (0.354–2.441)	0.875 (0.432–1.77)
Depth	<median	Reference			
	> = median	1.611 (0.778–3.337)	0.421 (0.196–0.905)*	1.612 (0.608–4.277)	1.079 (0.533–2.181)
Length-Width Ratio	<median	Reference			
	> = median	1.111 (0.539–2.291)	0.783 (0.375–1.631)	0.8 (0.302–2.122)	1.278 (0.631–2.586)
Length-Depth Ratio	<median	Reference			
	> = median	0.898 (0.435–1.852)	0.958 (0.462–1.986)	0.751 (0.283–1.991)	1.342 (0.663–2.716)
L/CS Ratio	<median	Reference			
	> = median	0.934 (0.452–1.93)	0.75 (0.355–1.581)	1.129 (0.429–2.971)	1.302 (0.638–2.658)
Volume	<median	Reference			
	> = median	1.491 (0.72–3.085)	0.606 (0.289–1.27)	1.512 (0.57–4.012)	0.875 (0.432–1.77)

**Fig. 3:**
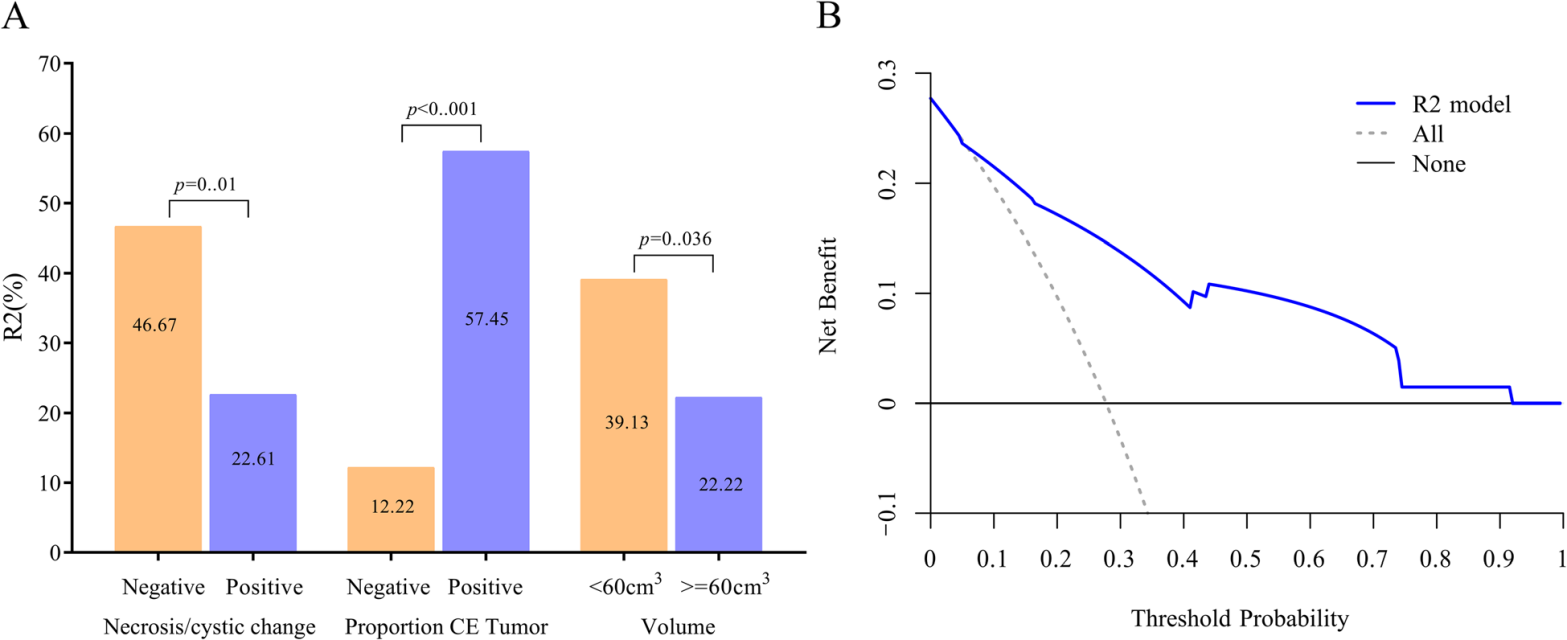
**a** Proportion of CE tumour (> 5%), volume < 60 cm^3^ and absence of necrosis/cystic change were associated with a significantly higher incidence of the R2 subtype. **b** Decision curve analysis demonstrated that prediction with the R2 model was clinically useful

**Fig. 4:**
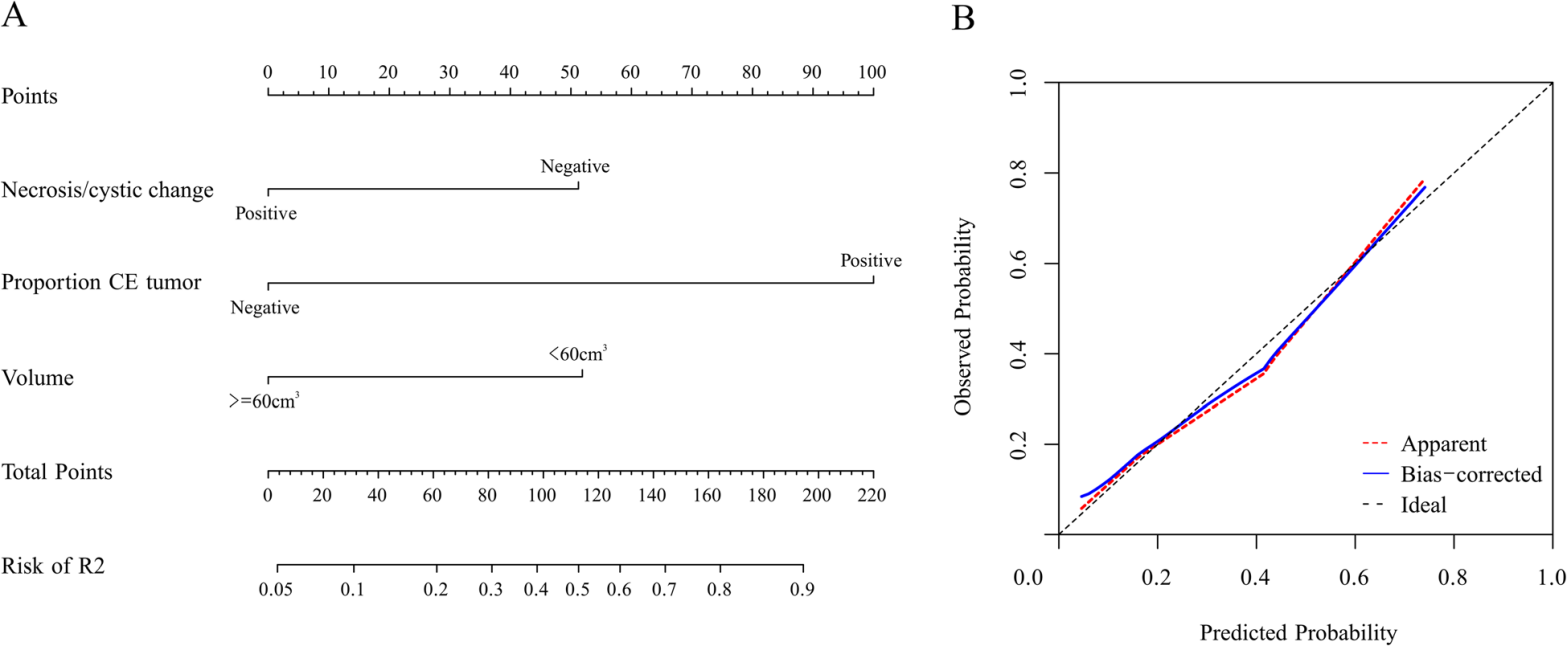
The nomogram (**a**) showed good discrimination and was well calibrated (**b**)

Multivariate analysis (Table 4) revealed that enhancing margin (ill-defined, OR: 9.985; *P* = 0.003) and T1 + C/T2 mismatch (yes, OR: 0.091; *P* = 0.023) were associated with the R1 subtype (AUC: 0.708). The average accuracy of the ten-fold cross validation was 71%. Proportion of contrast-enhanced (CE) tumour (> 5%, OR: 14.733; *P* < 0.001) and necrosis/cystic changes (yes, OR: 0.252; *P* = 0.009) were associated with the R2 subtype (Fig. 3a AUC: 0.832). The average accuracy of the ten-fold cross validation was 82%. Decision curve analysis (Fig. 3b) demonstrated that prediction with the R2 model was clinically useful. For the prediction of the R2 subtype, the nomogram showed good discrimination and calibration (Fig. 4). Haemorrhage (yes, OR: 8.55; *P* < 0.001) was positively associated with the R3 subtype (AUC: 0.689). The average accuracy of the ten-fold cross validation was 87%. Proportion of CE tumour (> 5%, OR: 0.14; *P* < 0.001) was negatively associated with the R4 subtype (AUC: 0.672). The average accuracy of the ten-fold cross validation was 71%.

**Table 4:** Results from risk analyses (multivariate logistic regression, odds ratios, 95% confidence intervals in parentheses)

		R1	*P*	R2	*P*	R3	*P*	R4	*P*
Necrosis/cystic	Negative			Reference					
	Positive			0.252 (0.090–0.709)	0.009				
Hemorrhage	Negative					Reference			
	Positive					8.55 (2.906–25.158)	< 0.001		
Proportion CE Tumor	Negative			Reference				Reference	
	Positive			14.733 (5.364–40.464)	< 0.001			0.14 (0.046–0.423)	< 0.001
Enhancing Margin	Well-defined	Reference							
	Poorly-defined	9.895 (2.218–44.134)	0.003						
T1 + C/T2 Mismatch	Negative	Reference							
	Positive	0.091 (0.012–0.720)	0.023						
Volume	<60cm^3^			Reference	0.006				
	> = 60 cm^3^			0.248 (0.091–0.675)

**Fig. 5:**
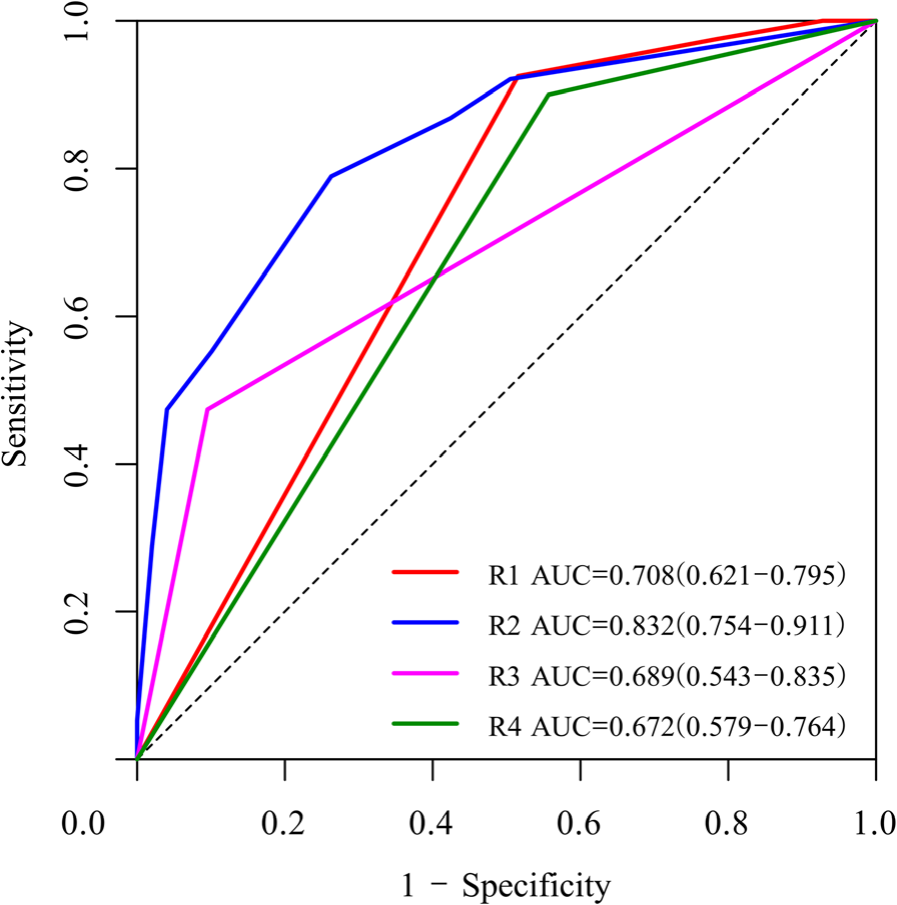
ROC analyses of each mRNA-based subgroup

We demonstrated (Fig. 5) that ill-defined margins and the absence of T1 + C/T2 mismatches were positively linked with the R1 subtype (AUC: 0.708). Proportion CE tumour (> 5%), volume < 60 cm^3^ and absence of necrosis/cystic change were positively associated with the R2 subtype (AUC: 0.832). Haemorrhage was positively associated with the R3 subtype (AUC: 0.689). Proportion of CE tumour > 5% was negatively associated with the R4 subtype (AUC: 0.672).

## Discussion

Glioma is one of the most common primary central nervous system malignant tumours [24, 25]. Intratumoural genetic heterogeneity plays a pivotal role in driving disease progression and therapeutic resistance in LGG. Intratumoural heterogeneity has been linked to metastatic potential and is likely to be an important prognostic feature of human cancer [26, 27]. The TCGA Research Network [17] classifies LGG into four subgroups (R1–R4) based on mRNA expression. R1, R2, R3 and R4 tumours were found to be biologically and clinically distinct. Our previous published work has revealed that clinical and MR features may therefore be used to facilitate the preoperative prediction of LGG IDH/1p19q subtype. In this research, we revealed that MRI features can identify distinct LGG mRNA-based molecular subtypes.

Radiogenomic studies have revealed key imaging differences between certain LGG genetic groups and may aid in the diagnosis of patients with LGG as well as predict survival and guide treatment in patients with LGG [10, 28–31]. In this study, R2 tumours showed significantly worse overall survival than the other RNA subtypes (R1 subtype, R3 subtype and R4 subtype), which did not significantly differ from one another. The time-dependent ROC for the R2 subtype was 0.78 for survival at 12 months, 0.76 for survival at 24 months, and 0.76 for survival at 36 months.

The R2 subtype is mostly composed of GIII tumours (77%), tumours mostly of astrocytoma histology (68%), tumours enriched for the methylation subtype M2 (62%) and IDH wild type (67%) tumours. This subtype is correlated with GBM-related events such as PTEN mutation, chromosome 10 loss, and EGFR mutation and amplification. Our findings showed that the proportion of CE tumours (> 5%) and the absence of necrosis/cystic changes were positively associated with the R2 subtype (AUC: 0.832). This is the first article to show the connection between the R2 subtype and MR features. For the prediction of the R2 subtype, the nomogram showed good discrimination and calibration. Decision curve analysis demonstrated that prediction with the R2 model was clinically useful.

The other RNA subtypes (R1, R3, and R4) were populated with IDH-mutant gliomas. R1 lacked 1p/19q codeletion and was comprised of two methylation subtypes, M5 (70%) and M3 (30%), and the vast majority of R1 cases had TP53 and ATRX mutations17. We demonstrated that well-defined margins and the absence of T1 + C/T2 mismatches were positively associated with the R1 subtype (AUC: 0.708). The R3 subtype was entirely composed of IDHmut-codeletion gliomas and was equally distributed across the methylation subtypes M2 and M3. It was also enriched for oligodendrogliomas (85%), mutations in NOTCH1, FUBP1, and CIC, and oligodendrocyte progenitor-specific expression. Our findings showed that haemorrhage was positively associated with the R3 subtype (AUC: 0.689). The R4 subtype highly expressed a neuron-specific signature and a neuroblastic astrocytoma signature. The proportion of CE tumours (<=5%) was positively associated with the R4 subtype (AUC: 0.672). This is the first article to show that MRI features can identify distinct LGG mRNA-based molecular subtypes (R1, R3–4). Radiogenomics analysis allows researchers to explore the TCGA and TCIA databases for correlations between mRNA-based molecular subtypes and radiological phenotypes.

Our study has several limitations. The major limitation of this article was that the sample size of the R3 subtype was only 19 patients (11.0%). The disadvantages of a small sample size might have limited the statistical power to explore additional correlations of the R3 subtype. Our findings should be further investigated and externally validated in larger cohorts of LGG patients. In addition, the MR data are heterogeneous, and in most cases, the images were acquired as part of routine care and not as part of a controlled research study or clinical trial. Our results should be validated using standardized MR imaging.

## Conclusions

Our results revealed connections between LGG mRNA-based subtypes (R1-R4) and MR lesion features. Our findings revealed that ill-defined margins and the absence of T1 + C/T2 mismatches were positively associated with the R1 subtype. The proportion of CE tumour > 5%, volume < 60 cm^3^ and absence of necrosis/cystic changes were positively associated with the R2 subtype. Haemorrhage was positively associated with the R3 subtype. Proportion of CE tumour > 5% was negatively associated with the R4 subtype (AUC: 0.672).

## Data Availability

No administrative permissions were required to access the raw data from The Cancer Genome Atlas Low Grade Glioma (TCGA-LGG) project. Public access to the databases is open. All data generated or analysed in this study are included in this published article. TCIA: https://wiki.cancerimagingarchive.net/; TCGA: https://portal.gdc.cancer.gov/.

## Abbreviations

LGG: Lower-grade glioma
CI: Confidence interval
OR: Odds ratio
TCIA: The Cancer Imaging Archive
CE: Contrast-enhanced
OS: Overall survival
AUC: Area under the receiver operating characteristic curve
TCGA: The Cancer Genome Atlas
SVZ: Subventricular zone
CS: The shortest distance between the lateral edge and the tumour centroid
